# Non-contrast Enhancing Group A Streptococcus Subdural Empyema: An Illustrative Case Report of a Potential Radiographic Pitfall

**DOI:** 10.7759/cureus.40623

**Published:** 2023-06-19

**Authors:** Youngkyung Jung, Carolyn Lai

**Affiliations:** 1 Neurosurgery, University of Toronto, Toronto, CAN; 2 Neurosurgery, Sunnybrook Health Sciences Centre, Toronto, CAN

**Keywords:** rim-enhancement, empyema, subdural, group a streptococcus, otolaryngology

## Abstract

Subdural empyemas (SDEs) are an uncommon complication of intracranial infection, typically presenting as a hypodense collection with peripheral contrast enhancement. Herein, we report two rare cases of SDE without contrast enhancement, both secondary to group A streptococcus. The first is a 27-year-old previously healthy female, at 27 weeks of gestational age who presented with fever, headache, neurological decline, and blood cultures positive for gram-positive cocci. The second case is a previously healthy 48-year-old male who presented with left-sided otalgia, fever, headache, and precipitous decline in altered mental status and hemiplegia. Computed tomography (CT) and magnetic resonance imaging (MRI) in both cases showed a subdural collection without contrast enhancement and without diffusion restriction. Despite appearances similar to subdural effusion, because of a heightened suspicion due to clinical decline, both were taken to surgery for irrigation and debridement which confirmed SDE. Both patients were treated with six weeks of intravenous antibiotics and eventually recovered without neurological deficits. SDEs are uncommon but clinically significant phenomena. These two cases demonstrate that SDEs in rare circumstances may present as non-enhancing subdural collections. Missing the diagnosis of SDE can have significant consequences to patient morbidity and mortality and as such, it not be excluded based on radiographic findings alone.

## Introduction

Subdural empyemas (SDE) are neurosurgical emergencies where delays in diagnosis and treatment can lead to significant neurologic sequelae, morbidity, and mortality. The etiology for these lesions remains variable, including contiguous spread from an ear/sinus infection, hematogenous spread from a more distal source, post-traumatic, and iatrogenic. Regardless, the mainstay for treatment includes surgical drainage for source control and targeted antibiotic therapy. Developments in neuroimaging and antimicrobial therapy have helped decrease the mortality rate of SDE to 6-15%, albeit with significant long-term morbidity including seizures and weakness/hemiparesis [1].

Radiographically, SDEs present as hypo or isodense collections with rim enhancement, which reflects the development of inflammatory granulation tissue [2]. Here, we illustrate two atypical cases of SDEs presenting as non-enhancing subdural collections in the context of group A streptococcus (GAS) bacteremia.

To our knowledge, only one other case of non-contrast enhancing SDE has been reported in the literature [3]. We aim to raise awareness that in occasional circumstances, SDEs can appear like subdural collections or effusions without enhancement. A raised index of suspicion for SDE in the appropriate clinical context is necessary to prevent the treatable morbidity of this neurosurgical emergency.

## Case presentation

Case 1

We present the case of a previously healthy 27-year-old female, with no history of immunocompromise, at 27 weeks of gestational age. She arrived at the hospital with several days of headaches, fever, and jaw pain, with acute exacerbation of symptoms within 24 hours. She was treated initially at a peripheral hospital but transferred for neurologic deterioration as she became progressively lethargic with a Glasgow Coma Scale (GCS) of 9 (E2V2M5) with significant aphasia, right-sided upper and lower extremity weakness on arrival to our hospital. Her white blood cell count was 18, and lactate 3, in keeping with a systemic infectious process. Of note, prior to this admission, she had contracted COVID-19 three months ago and had subsequently recovered with a monoclonal antibody treatment.

**Figure 1:**
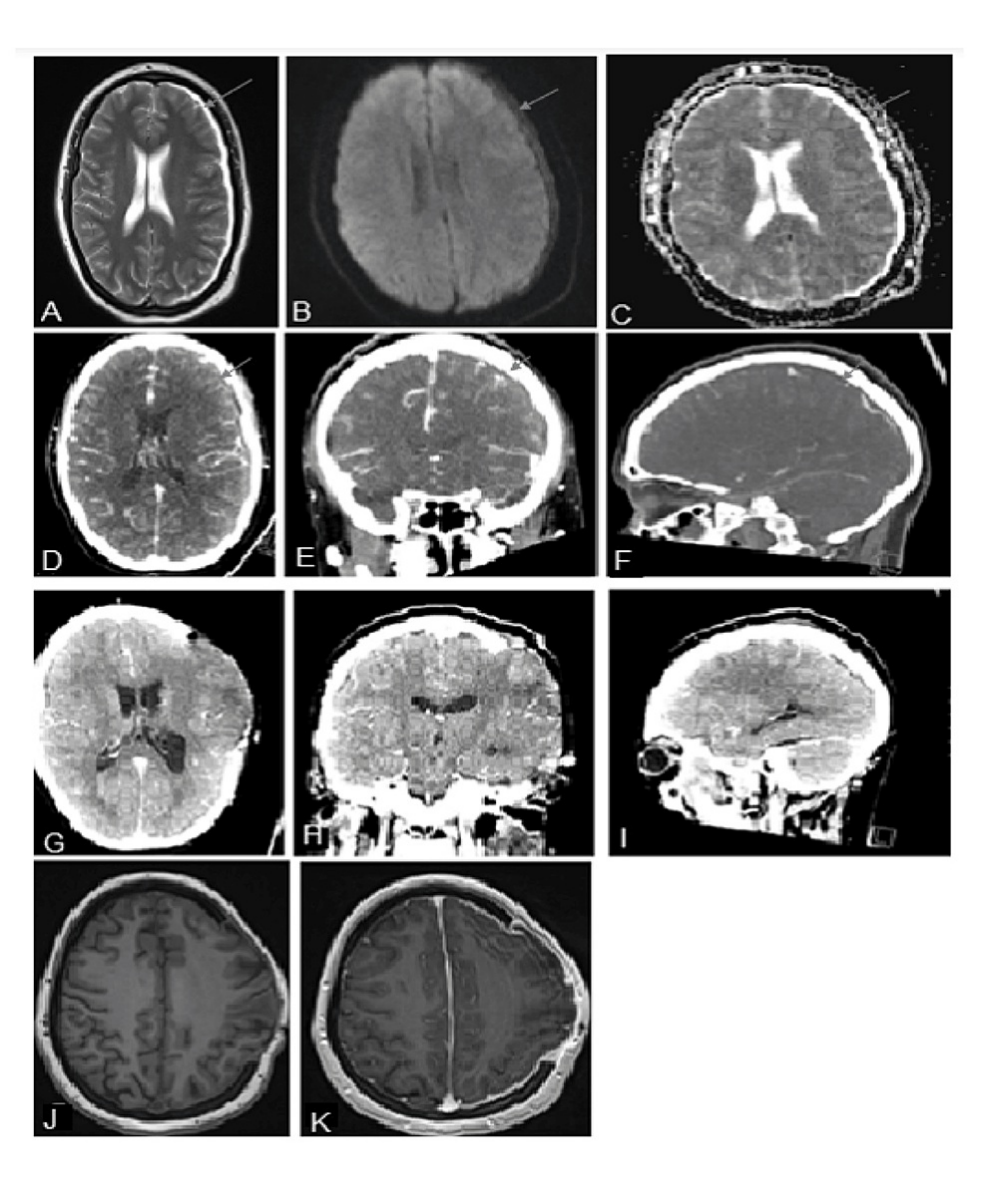
Radiographic images of a non-enhancing subdural empyema in a previously healthy 27-year-old female (A-C) preoperative axial view of a T2 weighted MRI demonstrating a left-sided subdural collection (A), with no diffusion restriction on DWI (B) and ADC (C) sequences. Hypodense left-sided subdural collection is outlined with a blue arrow. (D-F) Axial, coronal, and sagittal views of a preoperative contrast-enhanced CT head demonstrating a non-enhancing left-sided 2 mm hypodensity, outlined with a blue arrow. (G-I) Axial, coronal, and sagittal views of a postoperative contrast-enhanced CT head, two days post-op, demonstrating a left-sided decompressive craniectomy and interval resolution of the previous subdural collection. (J-K) Axial views of an unenhanced (J) and enhanced (K) T1 weighted MRI, obtained seven weeks after surgery, demonstrating interval resolution of the previous subdural collection with no enhancement in the surgical bed. MRI: magnetic resonance imaging; DWI: diffusion-weighted imaging; ADC: apparent diffusion coefficient; CT: computed tomography

An initial magnetic resonance image (MRI) of the head was consistent with a left-sided subdural collection, with no restriction diffusion (Figure 1A-1C); gadolinium was avoided due to its teratogenic effects. Initially, efforts were made to avoid a computed tomography (CT) scan due to the effects of radiation and gadolinium, however, due to ongoing clinical fluctuation, a subsequent CT head with contrast was consistent with a non-enhancing subdural collection (Figure 1D-1F). An initial lumbar puncture was unsuccessful, however, preliminary blood cultures were positive for gram-positive cocci in chains. Due to a high index of suspicion given her clinical deterioration, she was urgently booked for a left craniectomy for evacuation of presumed SDE where purulent contents were confirmed intraoperatively (Figure 1B-1F). Since imaging was inconsistent with an SDE, a large incision was planned but only a small portion and burr hole were opened initially. Once purulent SDE was confirmed, the remainder of the craniotomy was subsequently opened. The bone flap was left off as the brain continued to be swollen intraoperatively and there was intraoperative suspicion of early thrombosis. Final blood cultures and intraoperative cultures were positive for *Streptococcus pyogenes*, for which she was placed on a six-week course of penicillin G 4 million units IV q4h. Given the severity of her systemic illness, she underwent an urgent cesarean section the next day after craniotomy. Fortunately, her baby was well. After a couple of weeks of ICU stay, she improved neurologically and was eventually discharged for rehabilitation. At 18 months follow-up, she has recovered fully from her aphasia and hemiplegia without any speech deficits, weakness, or cognitive deficits. She underwent a cranioplasty, which was well-tolerated without complications. Her (now) infant also remains well.

Case 2

**Figure 2:**
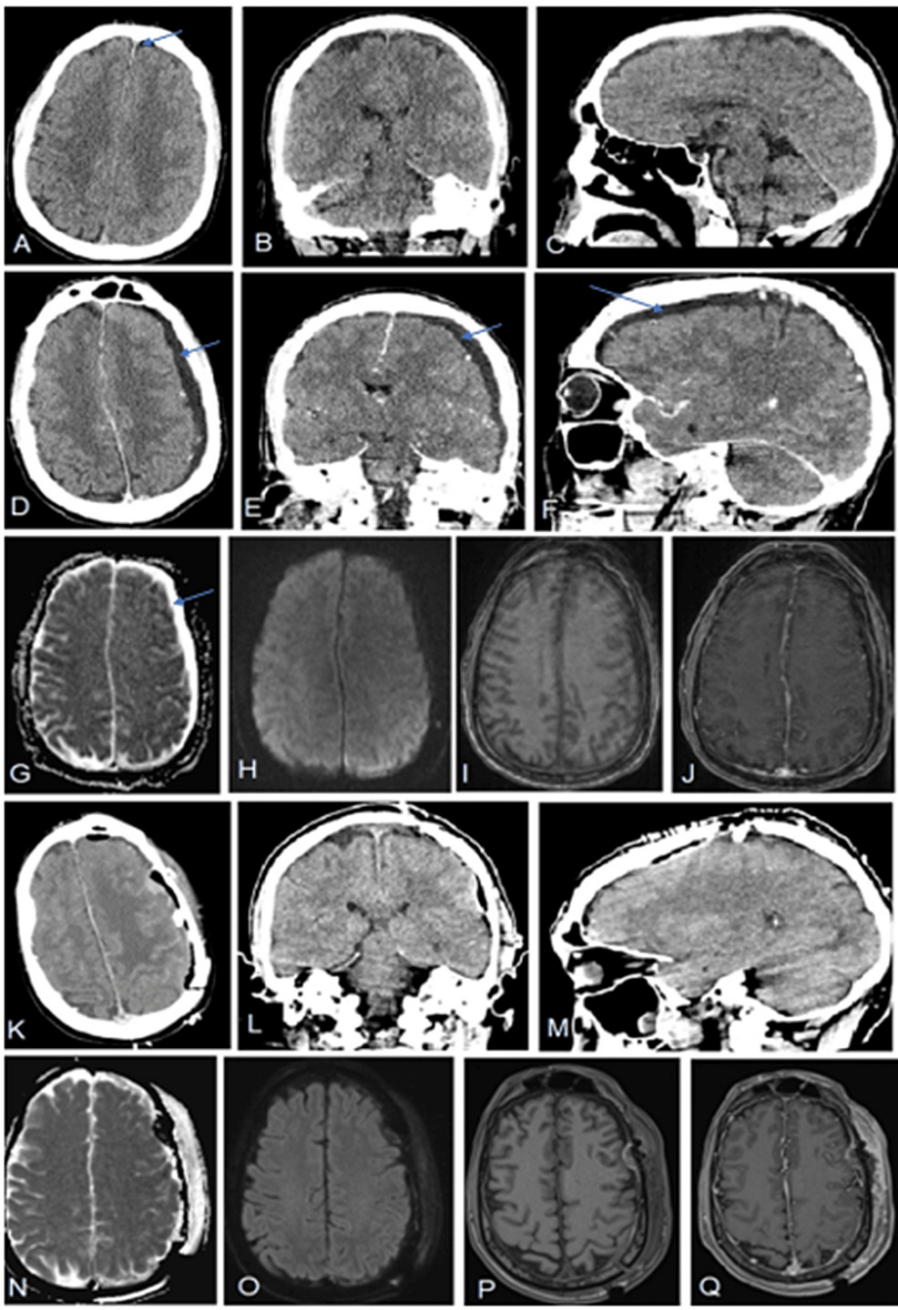
Radiographic images of a non-enhancing subdural empyema in a previously healthy 48-year-old male Figure 2: Each row, left to right reflects axial, coronal, and sagittal images. (A-C) Initial contrast-enhanced CT head demonstrating subtle left-sided hypodensities and trace pneumocephalus (blue arrow). (D-F) Repeat contrast-enhanced CT scan 72h later demonstrates interval development of a non-enhancing 5 mm left-sided hypodensity with significant mass effect (blue arrow). (G-J) Preoperative MRI; axial ADC (G) and DWI (H) sequences demonstrating a lack of diffusion restriction and T1-weighted axial images, without (I) and with gadolinium (J), demonstrating a lack of enhancement. (K-M) Postoperative CT head with contrast demonstrating interval left-sided craniotomy for subdural empyema evacuation. (N-Q) Postoperative MRI obtained five days later demonstrates a lack of diffusion restriction (N, O), overall improvement of mass effect, and no contrast enhancement in the surgical bed (P, Q). MRI: magnetic resonance imaging; DWI: diffusion-weighted imaging; ADC: apparent diffusion coefficient; CT: computed tomography

This second patient is a previously healthy, immunocompetent 48-year-old male who presented to an outside hospital with confusion, fever, and several days of left-sided otalgia and discharge from the left ear. Given concerns of meningitis, a lumbar puncture was done, positive for gram-positive cocci. He was treated with ceftriaxone, and an initial CT head demonstrated subtle left-sided hypodensities, but no significant subdural collection (Figure 2A-2C). He had an ongoing altered level of consciousness and symptoms of aphasia for which a repeat scan was done 24h later, demonstrating a significant non-enhancing left-sided subdural collection/effusion which radiologist commented was not empyema (Figure 2D-2F). Given his decline, he was transferred to our hospital, where within the time of transfer, he developed a worsening level of consciousness (LOC) and now right-sided hemiplegia. He was expeditiously intubated and taken for a left craniotomy for evacuation of presumed SDE, whilst our otolaryngology colleagues debrided the ear and placed a myringotomy tube in the left ear. Again, after a small opening intraoperatively confirmed purulent empyema, a larger craniotomy was performed to evacuate the SDE. Intraoperative cultures were consistent for GAS, for which he was placed on a six-week course of penicillin G. Postoperatively, he made significant gains, was extubated, and transitioned to acquired brain injury rehabilitation without focal deficits or aphasia.

## Discussion

Subdural collections and SDE are known complications of bacterial meningitis, occurring in approximately 2.7% of cases, and is a harbinger of poor neurological outcome [4]. Mechanisms of infection include direct seeding from an anatomically contiguous region (i.e., sinusitis) and seeding through valveless diploic veins. Both subdural effusions and SDE can be sequelae of meningitis. Subdural effusion can be common in bacterial meningitis, particularly in children, which are generally asymptomatic and tend to resolve spontaneously [5]. In contrast, SDE occurs more rarely in meningitis, is associated with rapid deterioration, and requires prompt surgical drainage [5]. Non-enhancing SDEs are an even rare phenomenon, the pathophysiology of which remains unclear. Although imaging may mimic subdural effusion, they should not be misdiagnosed as subdural effusion. Thus far, there has been only one other documented case of *Streptococcus pyogenes* SDE presenting with a non-enhancing subdural collection on imaging [3].

Presumably, the lack of enhancement reflects a lack of granular or inflammatory tissue to take up the contrast, although the reason for this in two young immunocompetent individuals is unclear. By corollary, intracranial abscess formation occurs in four distinct stages: early cerebritis, late cerebritis, early capsule formation, and late capsule formation. Although early cerebritis (occurs days 1-3 after initial infectious insult) can be associated with patchy to no enhancement on imaging, patients rarely present this early in the infectious process, and furthermore tend to be associated with significant perilesional edema, which was not seen in either case (Figure 1 and Figure 2) [6,7]. Perhaps in our two non-enhancing cases of SDE, they were detected at an earlier stage of SDE akin to cerebritis for intracranial abscess formation.

Another consideration is the rarity of GAS infections of the central nervous system, accounting for approximately 0-3% of SDEs, depending on the case series [4,8]. In a brief review of the literature, there have only been a few documented cases of (enhancing) GAS SDE (Table 1) [9-12], each with a direct source/cause of infection.

**Table 1:** Literature search of group A streptococcus-related subdural empyema

Study	Patient demographics	Presenting symptoms
Guh and Monserrate 2005 [12]	42-year-old male, history of dyslipidemia	Fever, oliguria, neck and facial pain, otalgia, lethargy
Ulloa-Gutierrez et al., 2005 [13]	Previously healthy 3-month-old male	Varicella infection, fever, rash, seizures, lethargy
Viola et al., 2009 [11]	Previously healthy 5-year-old male	Febrile seizures, flu-like symptoms
Tovar et al., 2019 [14]	Previously healthy 45-year-old female	Headache, left hemiparesis, seizures two weeks after epidural anesthesia prior to a cesarean section

The first case is remarkable due to the lack of a clear etiology. Other than a resolved case of COVID-19, there was no identifiable infectious source of infection. In two case reports, COVID-19 was associated with an SDE in patients with pre-existing sinusitis, raising concerns it may create a state of immunocompromise [13,14]. However, our patient had COVID-19 three months prior to presenting with an SDE, and with no other significant source of infection. The second case is atypical due to the delayed radiographic findings; he presented with a classic history of acute otitis media, meningitis, and symptoms suggestive of systemic infection, while the initial CT head demonstrated minimal subdural collections and trace pneumocephalus. One day later, a repeat CT head demonstrated significant growth of the subdural collection with subsequent mass effect. Viola et al. report a similar case of delayed radiographic findings of an SDE [11]. It remains unclear if this is due to host factors or remains a characteristic of GAS SDE.

Overall, these two cases demonstrate GAS SDEs may present as non-enhancing subdural collections, and that the diagnosis of SDE should ultimately be guided by clinical history and acumen.

## Conclusions

SDE are treatable neurosurgical emergencies, necessitating prompt recognition and intervention. Although SDEs classically present with peripherally enhancing collections on imaging with diffusion restriction, these two cases illustrate the importance of maintaining a raised index of suspicion for SDE in the appropriate clinical context.

Overall, we aim to illustrate that SDEs can, in rare circumstances, present as non-enhancing subdural collections, and radiographically mimic subdural effusions. They can occur even in healthy individuals. Timely recognition and management are key to minimizing morbidity and mortality.

Future studies and case tracking are needed to determine whether this phenomenon is unique to GAS or can be seen with other organisms and whether the lack of enhancement is associated with clinical outcomes.
